# Preoperative embolisation of brain arteriovenous malformations: a systematic review and meta-analysis

**DOI:** 10.1007/s10143-022-01766-8

**Published:** 2022-03-09

**Authors:** Conor Brosnan, Michael Amoo, Mohsen Javadpour

**Affiliations:** 1 National Neurosurgical Centre, Beaumont Hospital, Dublin 9, D09 V2N0 Ireland; 2 Royal College of Surgeons in Ireland, Dublin 2, Ireland; 3 Trinity College Dublin, Dublin 2, Ireland

**Keywords:** Embolisation, Arteriovenous malformation, Brain, Cerebral, Surgery, Preoperative

## Abstract

Preoperative embolisation is a commonly performed adjunct to microsurgical excision of brain arteriovenous malformations (bAVMs), with aims such as lessening the technical difficulty of the microsurgical procedure, reducing operative time, decreasing blood loss, and improving patient functional outcomes. We aim to perform a systematic review of randomised trials and cohort studies evaluating preoperative embolisation of bAVMs published between 01 January 2000 and 31 March 2021 and appraise its role in clinical practice. A MEDLINE search was performed, and articles reporting on outcomes following preoperative embolisation, as an adjunct to microsurgery, were eligible for inclusion. PRISMA reporting and Cochrane Handbook guidelines were followed. The primary outcome measure was the risk of complications associated with preoperative embolisation. The study was registered with PROSPERO (CRD42021244231). Of the 1661 citations, 8 studies with 588 patients met predefined inclusion criteria. No studies specifically compared outcomes of surgical excision of bAVMs between those with and without preoperative embolisation. Spetzler Martin (SM) grading was available in 301 cases. 123 of 298 (41⋅28%) patients presented with haemorrhage. Complications related to embolisation occurred in 175/588 patients (29.4%, 95% CI 19.6–40.2). Permanent neurological deficits occurred in 36/541 (6%, 95% CI 3.9–8.5) and mortality in 6/588 (0.41%, 95% CI 0–1.4). This is the first systematic review evaluating preoperative embolisation of bAVMs. Existing studies assessing this intervention are of poor quality. Associated complication rates are significant. Based on published literature, there is currently insufficient evidence to recommend preoperative embolisation of AVMs. Further studies are required to ascertain if there are benefits of this procedure and if so, in which cases.

**Supplementary Information:**

The online version contains supplementary material available at 10.1007/s10143-022-01766-8.

## Introduction

Brain arteriovenous malformations (bAVMs) are an uncommonly encountered phenomenon. Defined as congenital vascular abnormalities characterised by complex aggregations of tortuous intracranial arteries and veins, lacking intervening capillary beds [24], bAVMs were first described by Steinheil in 1895, while the first complete resection was performed by Cushing in 1929 [5]. They have an annual incidence rate of approximately 1 per 100,000 [1]. The annual haemorrhage rate in patients with unruptured bAVMs is 1–4% [6, 21]. Consequences of rupture may be devastating.

Numerous treatment strategies for bAVMs exist. Management varies from conservative approaches (medical treatment of symptoms only) to interventions aimed at eradicating the bAVM [18]. Microsurgical resection, endovascular therapy, and stereotactic radiosurgery may be used in isolation or in combination for eradication. Embolisation represents a relatively novel approach, its use first described only 60 years ago [16]. Many centres routinely utilise preoperative embolisation in advance of surgery [11, 26, 28]. “Eased handling” is a purported advantage of this, with reported reductions in size and flow through bAVM streamlining surgery [9]. Despite theorised advantages, there remains limited evidence to support the use of preoperative embolisation [19]. In addition, the practice comes with potential serious complications including stroke, permanent neurological disability and mortality.

To our knowledge, there are no published systematic reviews evaluating utilisation of preoperative embolisation for bAVMs. We have performed a detailed systematic review of available literature to evaluate the efficacy and complications of preoperative embolisation for bAVMs to help guide future practice.

## Materials and methods

This systematic review was performed following the *Cochrane Handbook for Systematic Reviews of Interventions *[11] and the Preferred Reporting Items for Systematic Reviews and Meta-Analyses (PRISMA) statement [13]. The study’s protocol was developed by authors CB and MJ and registered with the International Prospective Register of Systematic Reviews (PROSPERO), registration number: CRD42021244231 (https://www.crd.york.ac.uk/prospero/display_record.php?RecordID=244231).

### Search strategy

A review of the MEDLINE database was performed to identify randomised controlled trials (RCTs) and cohort studies evaluating preoperative embolisation of bAVMs. The search terms “Embolization”, “Embolisation”, “Onyx”, “Cryoacrylates”, “Ethylene vinyl alcohol copolymer”, “Ethylene–vinyl alcohol copolymer”, “Brain AVM”, “Brain arteriovenous malformation”, “Intracranial AVM”, “Intracranial arteriovenous malformation”, “Neurosurgery”, “Microsurgery”, “Preoperative”, “Pre-operative” and “Resection” were used in conjunction with Boolean operators “AND” and “OR”. All articles published between January 2000 and March 2021 were considered for inclusion. The final search date was 31 March 2021. Two independent reviewers (CB and MA) searched available literature. Initially, titles and then abstracts were screened. Full papers of suitable abstracts were reviewed. References from studies meeting inclusion and exclusion criteria were screened for eligibility.

### Eligibility criteria

Studies published in the English language explicitly evaluating preoperative embolisation in the management of bAVMs in all age groups were eligible for inclusion. Studies were only included if they exclusively reported outcomes (complication rates and/or functional outcomes) associated with the preoperative embolisation procedures, separate to microsurgical complications.

Studies with unclear methodology or those focusing on embolisation with curative intent or embolisation prior to radiosurgery were excluded. Studies were also excluded if the methodology failed to declare whether embolisation was performed as preoperative adjunct to microsurgery. Case reports and case series with less than 20 patients were excluded.

### Data extraction and statistical analysis

The following data were taken from selected publications: country, study type, year published, sample size, patient demographics (age and sex), Spetzler Martin (SM) grades, size and eloquent location, embolisation agent(s) used, complication rates related to embolisation (including the rate complications causing neurological deficits and the rate of permanent and transient neurological deficits), modified Rankin scale (mRS) score following preoperative embolisation and subsequently following microsurgical excision, mean blood loss from postembolisation surgical resection, rates of incomplete surgical resection and surgical complication rates. Data entered by two investigators (CB and MA) was compared following completion of review to attempt to eliminate selection bias. Duplicates were erased and discrepancies were resolved through review by the senior author (MJ).

All analysis was performed in *R v4.0.2* (The R Foundation for Statistical Computing, Vienna, Austria) using various packages [3, 30]. Binary outcomes were synthesised using random effects meta-analyses of proportions with inverse variance weighting. Variance was quantified by the standard deviation of the random effects, τ^2^, and was estimated using the DerSimonian-Laird method. Analysis was performed via the Freeman-Tukey double arcsine transformation as many studies had zero events, which was back-transformed to yield the summary measure [14]. The summary measure was the estimated proportion of each outcome, transformed to a percentage, with its corresponding 95% confidence interval.

Continuous outcomes were synthesised using random effects meta-analyses of means with the same weighting and variance estimation procedures. The means standard deviations for input were calculated directly from individual patient data provided by the case series in all analyses. Heterogeneity was quantified by the standard deviation of the random effects, τ^2^. We also described the impact of heterogeneity in terms of the proportion of heterogeneity not attributable to sampling variance, I^2^.

We hypothesised that the frequency of outcomes may be related to the SM grade of presenting AVMs. Hence, results are additionally described in subgroups of grades. We additionally fit mixed effects meta-regression models including the SM grade stratum (I + II, III or IV + V) to test this hypothesis, from which we describe the amount of heterogeneity accounted for by subgroups and the amount of residual heterogeneity using Cochran’s Q value.

### Primary and secondary outcomes

Complication rates related to preoperative embolisation alone (excluding the surgical complications) constitute the primary outcome of this study. Total complications of preoperative embolisation, haemorrhagic complications and transient and permanent neurological deficits were assessed. Secondary outcomes included reported functional outcomes directly following preoperative embolisation, complications of subsequent microsurgical excision and other measures of operative performance including blood loss, operative time, incomplete resection rates and subsequent post-resection functional outcomes.

### Methodological quality assessment

Quality assessment of included studies was performed by CB and MA separately using the National Institutes of Health (NIH) quality assessment tool for observational cohort and cross-sectional studies [23]. Studies were graded over fourteen separate major components with the response options “yes”, “no” and “cannot determine/not applicable/not reported” to each component. This allowed for stratification of studies as good, fair or poor quality. Authors discussed outcomes to resolve discrepancies.

### Role of the funding source

There was no funding source for this study.

## Results

**Fig. 1:**
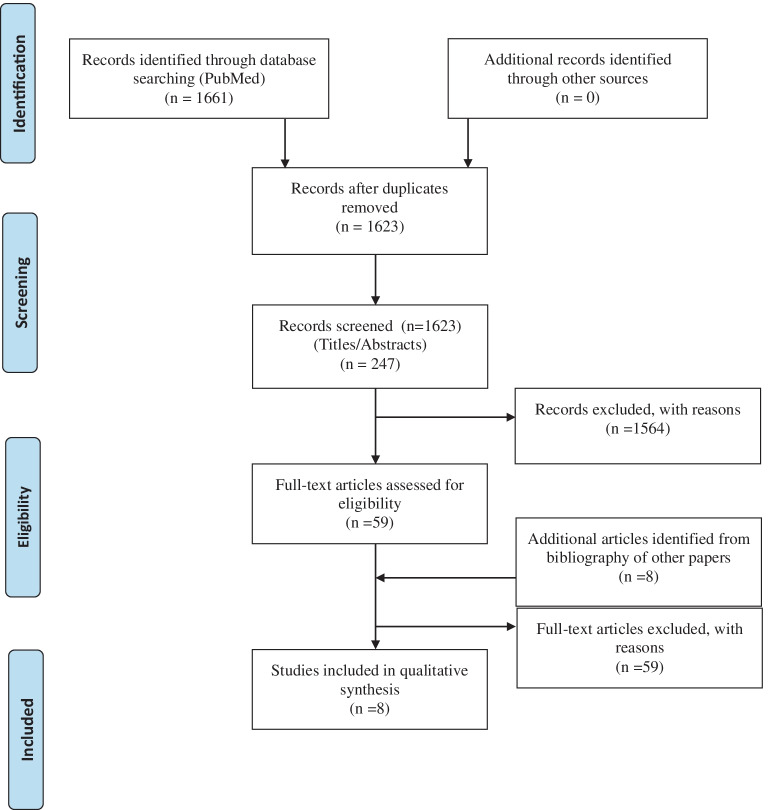
PRISMA statement for included studies in systematic review

In total, 1661 publications were found using the search criteria described in the “Materials and methods” section. 38 duplicates were removed. During screening, 1,564 publications met the predefined exclusion criteria. Of the remaining 59 studies, eight met the predefined inclusion criteria and were deemed to be of adequate quality to be included in the final review. These included seven retrospective cohort studies and one RCT. Included studies are listed in Table 1. A PRISMA statement is demonstrated in Fig. 1.

**Table 1:** Studies included in systematic review

Author	Country	Year	Journal	N patients	Design	Agent	SM grades	Conclusion
Taylor et al. [28]	Texas, USA	2004	J Neurosurg	201	Retrospective	Onyx/n-BCA/PVA/Coil	Patients not broken down by SM grades	Preoperative embolisation may reduce flow to AVM, intraoperative blood loss and operative time. Risks are not insignificant and must be considered
Steiger et al. [27]	Germany	2004	Acta Neurochir	38	Retrospective	Onyx/n-BCA	SM I + II:28, SM III:9, SM IV + V:2 Outcomes broken down according to SM grade	Intranidal embolisation prior to surgical resection of bAVM can lead to congested nidus and intraoperative bleeding. Delay should be considered with suspicion of congested residual nidus
Weber et al. [31]	Germany	2007	Neurosurgery	47	Retrospective	Onyx/n-BCA	SM I + II:25, SM III:10, SM IV + V:12 mRS broken down by SM grade	Preoperative onyx in bAVM treatment allows profound occlusion and provides a basis for safe resection
Natarajan et al. [20]	Washington, USA	2008	Neurosurgery	28	Retrospective	Onyx	SM I + II:13, SM III:8, SM IV + V:7 Outcomes broken down according to SM grade	Multimodality treatment with microsurgery is safe and effective
Hauck et al. [9]	Texas, USA	2009	Am J Neuroradiol	107	Retrospective	Onyx/n-BCA	SM I + II:17, SM III:15, SM IV + V:10 Results not broken down according to SM grades	Considerable risk for permanent neurologic deficit remains for bAVM embolisation. Risk must be carefully weighed against benefit
Loh et al. [15]	Washington, USA	2010	J Neurosurg	117	RCT	Onyx/n-BCA	SM I + II:61, SM III:33, SM IV + V:24 Results not broken down according to SM grades	Onyx is equivalent to n-BCA for safety and efficacy in preoperative embolisation of bAVM
Morgan et al. [19]	Australia	2013	J Neurosurg	538	Retrospective	Onyx/Other	Patients not broken down by SM grades	Outcomes for bAVM surgery not improved by embolisation. Embolisation did not reduce haemorrhagic complications
Luzzi et al. [17]	Italy	2018	World Neurosurg	27	Retrospective	Onyx	All patients SM grade III	Preoperative embolisation helped in surgical management of SM III bAVMs. Careful evaluation of angioarchitecture is required

**SM grades = **Spetzler-Martin Grades, **bAVM = **Brain arteriovenous malformation

Of the eight included studies, five were considered good quality according to the NIH quality assessment tool [15, 17, 19, 20, 31], and three were considered fair quality [9, 27, 28] (Table 2) All studies except one [15] randomised controlled trial (RCT) were retrospective non-randomised designs, which reduces our certainty in any meta-analytic findings. It is important to note that the one randomised trial^15]^ did not randomise patients to multiple interventions of interest and is thus effectively a prospective, single-arm cohort design for the purposes of our review. Quality ratings were predominantly downgraded because sample sizes were typically convenience samples rather than based upon prospective power analyses, retrospective design, outcome assessors were rarely blinded, and confounding variables were rarely adjusted for accurately. However, studies generally had clear objectives with a clear study population selected from the same population, outcomes and exposures were reliably measured, and there was low loss to follow-up. There was a substantial variation in geographic location, with four studies from the USA, two from Germany, and one from Australia and Italy. All studies were also single centre and thus may suffer from differences in baseline population, procedures, and policies and personnel expertise.

**Table 2:** Aggregated patient characteristics in the included studies

	Weighted mean (range)	*N* patients
Total patients		*N* = 588
Male: female	0⋅497:0⋅503 [9, 15, 17, 20, 28, 31]	*N* = 461
Mean age at presentation	37⋅57 (35⋅3–45⋅6) [9, 15, 17, 20, 28, 31]	*N* = 461
SM grade		
I + II	144 [9, 17, 20, 27, 31]	*N* = 301
III	102 [9, 17, 20, 27, 31]	*N* = 301
IV + V	55 [9, 17, 20, 27, 31]	*N* = 301
Size of AVM	3⋅65 cm (3⋅4–3⋅9 cm) [9, 27, 31]	*N* = 126
Eloquent area		
Yes	63⋅54% (50–75⋅61%) [9, 17, 20]	*N* = 96
No	36⋅46% (24⋅39–50%) [9, 17, 20]	*N* = 96
Presentation		
Rupture	41⋅28% (8⋅51–63⋅16%) [9, 17, 20, 27, 31]	*N* = 298
Other	58⋅72% (36⋅84–91⋅49) [9, 17, 20, 27, 31]	*N* = 298

Data from a total of 588 patients was evaluated from the eight studies included following literature review. All patients were treated with preoperative embolisation in advance of definitive surgical intervention. Six of the eight studies provided a breakdown of patients according to SM grade and mode of presentation [9, 15, 17, 20, 27, 31]. Forty-eight percent (144/301) of these patients had bAVMs of SM grade I + II, 34% (102/301) were SM grade III, and 18% (55/301) were SM grade IV and V. Of the patients, 41.28% (123/298) presented with a ruptured bAVM, and the other 58.72% (175/298) were unruptured. Detailed patient demographics are displayed in Tables 3 and 4.

**Table 3:** Quality assessment of included studies and overall quality ranking using the NIH quality assessment tool for observational cohort and cross-sectional studies

Study	1	2	3	4	5	6	7	8	9	10	11	12	13	14	Score	Quality rating
Taylor et al. [28]	Yes	Yes	Yes	No	Yes	Yes	Yes	NA	No	NA	Yes	NA	Yes	No	8	Fair
Steiger et al. [27]	Yes	Yes	Yes	Yes	No	Yes	Yes	NA	Yes	NA	No	NA	Yes	No	8	Fair
Weber et al. [31]	Yes	Yes	Yes	Yes	No	Yes	Yes	NA	Yes	NA	Yes	NA	Yes	No	9	Good
Natarajan et al. [20]	Yes	Yes	Yes	Yes	No	Yes	Yes	NA	Yes	NA	Yes	NA	Yes	No	9	Good
Hauck et al. [9]	Yes	Yes	Yes	Yes	Yes	Yes	Yes	NA	No	NA	No	NA	Yes	No	8	Fair
Loh et al. [15]	Yes	Yes	NA	Yes	Yes	Yes	Yes	NA	No	NA	Yes	NA	Yes	Yes	9	Good
Morgan et al. [19]	Yes	Yes	Yes	Yes	Yes	Yes	Yes	NA	Yes	NA	Yes	NA	Yes	Yes	11	Good
Luzzi et al. [17]	Yes	Yes	Yes	Yes	No	Yes	Yes	NA	Yes	NA	Yes	NA	Yes	No	9	Good

**Table 4:** Major components of the NIH quality assessment tool for observational cohort and cross-sectional studies

Major components
1. Was the research question/ objective in this paper clearly stated?
2. Was the study population clearly specified and defined?
3. Was the participation rate of eligible persons at least 50%?
4. Were all the subjects selected or recruited from the same or similar populations (including the same time period)? Were inclusion and exclusion criteria for being in the study prespecified and applied uniformly to all participants?
5. Was a sample size justification, power description, or variance and effect estimates provided?
6. For the analyses in this paper, were the exposure(s) of interest measured prior to the outcome(s) being measured?
7. Was the timeframe sufficient so that one could reasonably expect to see an association between exposure and outcome if it existed?
8. For exposures that can vary in amount or level, did the study examine different levels of the exposure as related to the outcome (e.g. categories of exposure, or exposure measured as continuous variable)?
9. Were the exposure measures (independent variables) clearly defined, valid, reliable, and implemented consistently across all study participants?
10. Was the exposure(s) assessed more than once over time?
11. Were the outcome measures (dependent variables) clearly defined, valid, reliable, and implemented consistently across all study participants?
12. Were the outcome assessors blinded to the exposure status of participants?
13. Was loss to follow-up after baseline 20% or less?
14. Were key potential confounding variables measured and adjusted statistically for their impact on the relationship between exposure(s) and outcome(s)?

The total complication rate following preoperative embolisation across all included studies was 175/588 (29.4%, 95% CI 19.6–40.2). This includes complications leading to permanent neurological deficits, transient neurological deficits and technical complications which did not manifest with any clinical deficits (Table 5). Overall, 62/588 (9.7%, 95% CI 5.6–14.6) patients had complications leading to neurological deficits, of which 36/541 (6%, 95% CI 3.9–8.5) were permanent and 16/452 (3%, 95% CI 1.4–5) were transient. Haemorrhagic complications occurred in 48/387 (12.6%, 95% CI 4.9–23). Mortality following preoperative embolisation occurred in 6/588 (0.4%, 95% CI 0–1.4). Forest plots showing the study level contributions to aggregate categorical outcomes are shown in Supplementary Fig. 1, while contributions to blood loss and operative time are shown in Supplementary Figs. 2 and 3, respectively.

**Table 5:** Aggregated findings from meta-analyses across all Spetzler-Martin grades and presentations

Complication	Studies	Events	*N* patients	% (95%CI)	*I*^2^	τ^2^
Complications of embolisation	8	175	588	29.4 (19.6–40.2)	84.9%	0.021
Leading to deficit	8	62	588	9.69 (5.63–14.6)	62.9%	0.0062
Permanent deficit	7	36	541	6 (3.88–8.48)	7.38%	0.00028
Transient deficit	6	16	452	2.99 (1.41–4.99)	0%	0
Technical without deficit	6	88	461	18.1 (7.69–31.4)	89.1%	0.03
Haemorrhagic	7	48	387	12.6 (4.87–23)	84.6%	0.026
Non-haemorrhagic	7	102	387	24.1 (8.64–43.8)	93.7%	0.071
mRS > 2 following embolisation	2	7	136	5.12 (1.12–11.3)	36.6%	0.0023
Mortality	8	6	588	0.409 (0–1.37)	0%	0
Subsequent surgery	4	29	182	16.5 (8.15–26.7)	59.5%	0.0089
Leading to deficit	4	17	182	6.63 (1.29–14.8)	62.5%	0.01
Permanent deficit	4	21	182	11.1 (6.69–16.3)	0%	0
Transient deficit	4	5	182	1.46 (0–9.45)	79.1%	0.023
Haemorrhagic	4	13	191	5.63 (1.98–10.6)	26.6%	0.0021
Non-haemorrhagic	3	11	144	6.77 (0–25.3)	87%	0.042
Residual AVM post-treatment	4	4	140	2.16 (0.0502–6.08)	11.8%	0.00096
Infection	2	1	55	1.17 (0–6.9)	0%	0
Mortality	5	6	229	1.27 (0–4.43)	36%	0.0032
mRS > 2 following surgery	4	26	191	12.7 (6.05–21.1)	52.5%	0.0063

*% (95%CI)* result from meta-analysis of proportions with 95% confidence interval in parentheses unless otherwise stated, *mRS* modified Rankin scale, *AVM* arteriovenous malformation

Moderate heterogeneity was observed for complications of both embolisation (*I*^2^ = 85%, *τ*^2^ = 0.02) and subsequent surgery (*I*^2^ = 59.5%, *τ*^2^ = 0.01). Meta-regression revealed that subgrouping by SM grades accounted for a substantial degree of heterogeneity in complications of both embolisation (*Q* = 18.52, *p* = 0.0003) and surgery (*Q* = 48, *p* < 0.0001). A small but significant amount of heterogeneity remained for complications of embolisation (residual *τ*^2^ = 0.09, *p* = 0.001), but no significant heterogeneity remained for complications of surgery (residual *τ*^2^ = 0.009, *p* = 0.16). Therefore, it appears that most of the heterogeneity was accounted for by dispersion across SM grades.

Four studies allowed for outcomes associated with specific SM grades to be extrapolated [17, 20, 27, 31]. Complication rates were calculated for 65 patients with SM grade I + II AVMs, 54 patients with SM grade III AVMs and 19 patients with SM grade IV + V AVMs (detailed outcomes for patients according to SM grade are provided in Tables 6, 7 and 8). Total complication rates of preoperative embolisation were calculated to be 7/40 (16.6%, 95% CI 5.5–31.3), 10/44 (22.8%, 95% CI 0–71.6) and 4/9 (43.9%, 95% CI 9–81.8) for SM grade I + II, SM grade III and SM grade IV + V bAVMs, respectively. Permanent neurological deficits occurred in 1/40 (1.8%, 95% CI 0–9.9), 4/44 (7.5%, 95% CI 0–29.5) and 1/9 (7.4%, 95% CI 0–41.5) of patients with SM grade I + II, SM grade III and SM grade IV + V bAVMs. Forest plots showing the study level contributions to outcomes per SM grade are shown in Supplementary Figs. 4, 5 and 6 for SM grades I, I + II and IV + V respectively.

**Table 6:** Aggregated findings from meta-analyses for Spetzler-Martin grades I and II

Complication	Studies	Events	*N* patients	% (95%CI)	*I*^2^	τ^2^
Complications of embolisation	2	7	40	16.6 (5.54–31.3)	8.14%	0.0012
Leading to deficit	2	1	40	1.76 (0–9.86)	0%	0
Permanent deficit	2	1	40	1.76 (0–9.86)	0%	0
Transient deficit	2	0	40	0 (0–4.48)	0%	0
Technical without deficit	2	6	40	14.5 (4.56–27.9)	0%	0
Haemorrhagic	2	7	40	16.6 (5.54–31.3)	8.14%	0.0012
Non-haemorrhagic	2	0	40	0 (0–4.48)	0%	0
mRS > 2 following embolisation	1	1	25	4 (0–16.4)	*N/A*	*N/A*
Mortality	3	0	65	0 (0–2.96)	0%	0
Subsequent surgery	2	4	40	10.4 (0–35.4)	67.7%	0.029
Leading to deficit	2	3	40	7.13 (0–21.9)	33.7%	0.007
Permanent deficit	2	2	40	4.55 (0–14.5)	0%	0
Transient deficit	2	1	40	1.51 (0–14.6)	49.6%	0.014
Haemorrhagic	1	1	13	7.69 (0–30.1)	*N/A*	*N/A*
Non-haemorrhagic	1	1	13	7.69 (0–30.1)	*N/A*	*N/A*
Residual AVM post-treatment	0	0	0	*N/A*	*N/A*	*N/A*
Infection	1	1	13	7.69 (0–30.1)	*N/A*	*N/A*
Mortality	3	1	65	0.414 (0–5.53)	13.9%	0.0019
mRS > 2 following surgery	2	1	38	1.57 (0–14.6)	46.6%	0.012
Blood loss (mean in ml)	2	*N/A*	34	263.5 (164.1–362.9)	0%	0
Operative time (mean in minutes)	2	*N/A*	34	268.7 (242.1–295.4)	0%	0

*% (95%CI)* result from meta-analysis of proportions with 95% confidence interval in parentheses unless otherwise stated, *mRS* modified Rankin scale, *AVM* arteriovenous malformation, *N/A* inestimable value

**Table 7:** Aggregated findings from meta-analyses for Spetzler-Martin grade III

Complication	Studies	Events	*N* patients	% (95%CI)	*I*^2^	τ^2^
Complications of embolisation	3	10	44	22.8 (0–71.6)	88.3%	0.15
Leading to deficit	3	6	44	12.6 (0–50.5)	82.8%	0.095
Permanent deficit	3	4	44	7.48 (0–29.5)	62.3%	0.033
Transient deficit	3	2	44	2.52 (0–20.6)	61.3%	0.031
Technical without deficit	3	4	44	7.01 (0.0954–19.6)	14.7%	0.0034
Haemorrhagic	3	5	44	9.16 (0–52.4)	87.4%	0.14
Non-haemorrhagic	3	5	44	9.18 (0.948–22.1)	9.95%	0.0022
mRS > 2 following embolisation	1	1	10	10 (0–38.1)	*N/A*	*N/A*
Mortality	4	0	54	0 (0–3.24)	0%	0
Subsequent surgery	3	5	44	10.6 (2.08–22.8)	0%	0
Leading to deficit	3	2	44	2.99 (0–12.1)	0%	0
Permanent deficit	3	2	44	2.99 (0–12.1)	0%	0
Transient deficit	3	0	44	0 (0–3.73)	0%	0
Haemorrhagic	2	0	35	0 (0–4.45)	0%	0
Non-haemorrhagic	2	0	35	0 (0–4.45)	0%	0
Residual AVM post-treatment	4	2	54	1.94 (0–9.32)	0%	0
Infection	2	0	35	0 (0–4.45)	0%	0
Mortality	4	0	54	0 (0–3.24)	0%	0
mRS > 2 following surgery	3	8	45	12.2 (0.53–31.7)	48%	0.018
Blood loss (mean in ml)	2	*N/A*	13	273.5 (98.99–448)	0%	0
Operative time (mean in minutes)	2	*N/A*	13	307.2 (111.8–502.5)	93.8%	18,632

*% (95%CI)* result from meta-analysis of proportions with 95% confidence interval in parentheses unless otherwise stated, ***mRS*** modified rankin scale, *AVM* arteriovenous malformation, ***N/A*** inestimable value

**Table 8:** Aggregated findings from meta-analyses for Spetzler-Martin grades IV and V

Complication	Studies	Events	*N* patients	% (95%CI)	*I*^2^	*τ*^2^
Complications of embolisation	2	4	9	43.9 (9.01–81.8)	0%	0
Leading to deficit	2	1	9	7.39 (0–41.5)	0%	0
Permanent deficit	2	1	9	7.39 (0–41.5)	0%	0
Transient deficit	2	0	9	0 (0–18.2)	0%	0
Technical without deficit	2	2	9	18 (0–55.8)	0%	0
Haemorrhagic	2	1	9	8.99 (0–77.7)	63.5%	0.12
Non-haemorrhagic	2	3	9	26.3 (0–73)	22.4%	0.019
mRS > 2 following embolisation	1	2	12	16.7 (0.412–43.9)	NA%	NA
Mortality	3	0	21	0 (0–6.76)	0%	0
Subsequent surgery	2	5	9	38.3 (0–100)	70.5%	0.16
Leading to deficit	2	5	9	38.3 (0–100)	70.5%	0.16
Permanent deficit	2	2	9	18 (0–55.8)	0%	0
Transient deficit	2	3	9	26.3 (0–73)	22.4%	0.019
Haemorrhagic	1	1	7	14.3 (0–51.7)	NA%	NA
Non-haemorrhagic	1	5	7	71.4 (31.8–99)	NA%	NA
Residual AVM post-treatment	0	0	0	N/A	N/A	N/A
Infection	1	0	7	0 (0–23.2)	NA%	NA
Mortality	3	0	21	0 (0–6.76)	0%	0
mRS > 2 following surgery	2	3	19	15.6 (1.43–37.1)	0%	0
Blood loss (mean in ml)	2*		13	858.3 (412.4–1304)	NA%	NA
Operative time (mean in minutes)	2*		13	372.5 (294.4–450.6)	NA%	NA

*% (95%CI)* result from meta-analysis of proportions with 95% confidence interval in parentheses unless otherwise stated, *mRS* modified Rankin scale, *AVM* arteriovenous malformation, *N/A* inestimable value, * one study excluded as *n* = 1 patients: heterogeneity therefore inestimable

Three studies allowed outcomes of preoperative embolisation to be compared between patients that presented with ruptured versus unruptured bAVMs [20, 27, 31]. Thirty-two patients presented with ruptured bAVMs, whilst 81 patients presented with unruptured bAVMs (detailed outcomes for patients according to mode of presentation are provided in Tables 9 and 10). A total of 6/28 (21%, 95% CI 7.1–38.9) patients with a ruptured bAVM suffered from complications after preoperative embolisation. Of these, 2/28 (4.7%, 95% CI 0–26.4) patients with ruptured bAVMs had post-embolisation neurological deficits, of which 2/28 (4.7%, 95% CI 0–26.4) were transient and none was permanent. Overall, 10/38 (24.9%, 95% CI 9.2–44.6) patients receiving treatment for unruptured bAVMs suffered from complications of embolisation. Of these patients, 3/38 (7.8%, 95% CI 0.6–19.4) had neurological deficits, all of which were permanent. Forest plots showing the study level contributions to outcomes per mode of presentation are shown in Supplementary Figs. 7 and 8 for ruptured and unruptured bAVMs, respectively.

**Table 9:** Aggregated findings from meta-analyses for outcomes in patients presenting with ruptured bAVMs

Complication	Studies	Events	*N* patients	% (95%CI)	*I*^2^	τ^2^
Complications of embolisation	2	6	28	21 (7.11–38.9)	0%	0
Leading to deficit	2	2	28	4.68 (0–26.4)	58.5%	0.024
Permanent deficit	2	0	28	0 (0–6.84)	0%	0
Transient deficit	2	2	28	4.68 (0–26.4)	58.5%	0.024
Technical without deficit	2	4	28	14.3 (2.83–30.7)	0%	0
Haemorrhagic	2	2	28	4.68 (0–26.4)	58.5%	0.024
Non-haemorrhagic	2	4	28	14.3 (2.83–30.7)	0%	0
mRS > 2 following embolisation	1	1	4	25 (0–79.3)	*N/A*	*N/A*
Mortality	3	0	32	0 (0–5.11)	0%	0
Subsequent surgery	2	5	28	16.7 (1.12–41.4)	49.6%	0.017
Leading to deficit	2	4	28	13.6 (2.47–29.9)	0%	0
Permanent deficit	2	2	28	7.14 (0.00996–21.1)	0%	0
Transient deficit	2	2	28	4.68 (0–26.4)	58.5%	0.024
Haemorrhagic	2	1	28	2.29 (0–13.4)	1.28%	0.00022
Non-haemorrhagic	2	3	28	7.11 (0–39)	75.2%	0.052
Residual AVM post-treatment	2	0	28	0 (0–6.84)	0%	0
Infection	2	0	28	0 (0–6.84)	0%	0
Mortality	3	1	32	0.922 (0–10.7)	0%	0
mRS > 2 following surgery	2	2	18	8.66 (0–29.5)	0%	0
Blood loss (mean in ml)	2	*N/A*	7	901.8 (0–1937)	0%	0
Operative time (mean in minutes)	2	*N/A*	7	332.9 (213.3–452.5)	0%	0

*% (95%CI)* result from meta-analysis of proportions with 95% confidence interval in parentheses unless otherwise stated, *mRS* modified Rankin scale, *AVM* arteriovenous malformation, *N/A* inestimable value

**Table 10:** Aggregated findings from meta-analyses for outcomes in patients presenting with unruptured bAVMs

Complication	Studies	Events	*N* patients	% (95%CI)	*I*^2^	τ^2^
Complications of embolisation	2	10	38	24.9 (9.15–44.6)	33.8%	0.007
Leading to deficit	2	3	38	7.75 (0.641–19.4)	0%	0
Permanent deficit	2	3	38	7.75 (0.641–19.4)	0%	0
Transient deficit	2	0	38	0 (0–4.87)	0%	0
Technical without deficit	2	7	38	16.6 (3.09–36.2)	41.9%	0.0099
Haemorrhagic	2	9	38	19.9 (1.24–49.4)	70.5%	0.033
Non-haemorrhagic	2	1	38	1.55 (0–13.5)	41.6%	0.0098
mRS > 2 following embolisation	1	3	43	6.98 (0.911–16.9)	*N/A*	*N/A*
Mortality	3	0	81	0 (0–2.18)	0%	0
Subsequent surgery	2	8	38	21.8 (3.57–47.7)	61.6%	0.022
Leading to deficit	2	7	38	18.3 (5.13–36.1)	28.9%	0.0056
Permanent deficit	2	5	38	13 (3.42–26.4)	0%	0
Transient deficit	2	2	38	3.86 (0–27)	72.9%	0.037
Haemorrhagic	2	3	38	7.75 (0.641–19.4)	0%	0
Non-haemorrhagic	2	5	38	13.4 (0–44)	75.5%	0.042
Residual AVM post-treatment	2	1	38	1.55 (0–13.5)	41.6%	0.0098
Infection	2	1	38	1.55 (0–13.5)	41.6%	0.0098
Mortality	3	0	81	0 (0–2.18)	0%	0
mRS > 2 following surgery	2	3	57	4.69 (0.186–12.7)	0%	0
Blood loss (mean in ml)	2	*N/A*	53	332.6 (171.6–493.7)	71.2%	9759
Operative time (mean in minutes)	2	*N/A*	53	288.8 (261.1–316.5)	0%	0

*% (95%CI)* result from meta-analysis of proportions with 95% confidence interval in parentheses unless otherwise stated, *mRS* modified Rankin scale, *AVM* arteriovenous malformation, *N/A* inestimable value

Three studies reported functional outcomes following preoperative embolisation. Morgan et al. and Weber et al. assessed functional outcomes using modified Rankin scale (mRS) scores [19, 31]. Seven of 136 (5.1%, 95% CI 1.1–11.3) patients had a mRS score of > 2 following preoperative embolisation [19, 31]. Loh et al. measured clinical outcome according to the Barthel Index (BI) with 21.37% of patients declining clinically following embolisation [12]. Weber et al. provided a patient-by-patient breakdown of mRS scores at time of presentation and post embolisation. In this study, 23.4% (11/47) patients had a higher mRS score following embolisation, with almost half of these (4/47) developing an mRS score > 2. Weber et al. also provided mRS scores for AVMs with different SM grades. Functional outcomes were found to be generally worse in patients treated with bAVMs of higher SM grade. Four percent (1/25) of those with SM grade I + II AVMs had an mRS score of > 2 following preoperative embolisation, whilst 10% (1/10) of those with SM grade III and 16.67% (2/12) of those with SM grade IV + V AVMs had an mRS score > 2. Weber et al. provided sufficient data to allow mRS scores to be differentiated between patients that had presented with haemorrhage and those who had not. They demonstrated that 25% (1/4) of patients with ruptured bAVMs had an mRS score > 2 whilst 6.98% (3/43) of patients treated with unruptured bAVMs had an mRS score > 2 following preoperative embolisation.

Five studies provided information on subsequent surgical intervention following preoperative embolisation [17, 19, 20, 27, 31]. The total complication rate associated with definitive surgical intervention after preoperative embolisation was 29/182 (16.5%, 95% CI 8.2–26.7). Permanent neurological deficits were calculated to occur at a rate of 21/182 (11.1%, 95% CI 6.7–16.3) following surgery. Transient neurological deficits occurred in 5/182 (1.5%, 95% CI 0–9.5). Haemorrhagic complications occurred in 13/191 (5.6%, 95% CI 2–10.6). Mortality rate was 6/229 (1.3%, 95% CI 0–4.4).

Complication rates of surgical excision following preoperative embolisation were higher in those with higher SM grade AVMs [17, 20, 27, 31]. Four of forty (10.4%, 95% CI 0–35.4) patients with SM grade I + II AVMs suffered from surgical complications, with 3/40 (7.1%, 95% CI 0–21.9) patients suffering a neurological deficit. Of these, 2/40 (4.6%, 95% CI 0–14.5) were permanent. Five of forty-four (10.6%, 95% CI 2.1–22.8) of those with SM grade III AVMs suffered from complications, with 2/44 (3%, 95% CI 0–12.1) patients having neurological deficits, all of which were permanent. In patients with SM grade IV + V AVMs, 5/9 (38.3%, 95% CI 0–100) suffered from complications following surgery. All of these led to neurological deficits and 2/9 (18%, 95% CI 0–55.8) patients were left with a permanent neurological deficit.

Complication rates from surgery varied depending on clinical presentation. Five of twenty-eight (16.7%, 95% CI 1.1–41.4) patients with ruptured bAVMs suffered from complications following surgery. Four of twenty-eight (13.6%, 95% CI 2.5–30) patients suffered from neurological deficits, of which 2 were permanent (7.1%, 95% CI 0.01–21.1). Eight of thirty-eight (21.8%, 95% CI 3.6–47.7) of those with unruptured bAVMs suffered from complications; of these, 7/38 (18.3%, 95% CI 5.1–36.1) resulted in deficits, 5 of which were all permanent (13%, 95% CI 3.42–26.4).

Twenty-six of 191 (12.7%, 95% CI 6.1–21.1) patients were reported to have an mRS score of > 2 following definitive surgery at the time of last clinical follow-up. Patients treated with higher SM grade bAVMs had higher rates of significant disability at follow up. One of thirty-eight (1.6%, 95% CI 0–14.6) patients with SM grade I + II bAVMs, 8/45 (12.2%, 95% CI 0.5–31.7) patients with SM grade III bAVMs and 3/19 (15.6%, 95% CI 1.4–37.1) patients with SM grade IV + V bAVMs had mRS scores > 2 at the time of their clinical follow-up. Two of eighteen (8.7%, 95% CI 0–29.5) patients presenting with ruptured bAVMs had an mRS score > 2 postoperatively compared to 3/57 (4.7%, 95% CI 0.2–12.7) of those presenting with unruptured bAVMs.

An incomplete resection rate of 4/140 (2.2%, 95% CI 0.05–6.1) was calculated having been explicitly mentioned by three studies [17, 20, 31]. A mean operative time of 5.79 h (*n* = 229) and mean blood loss of 680.28 ml (*n* = 257) were recorded from available data. Operative time was found to be longer in patients with higher grade AVMs, ranging from 268.7 min (95% CI 242–295) in SM grade I + II to 307.2 min (95% CI 111.8–502.5) in SM grade III and 372.5 min (95% CI 294–450.6) in SM grade IV + V AVMs). Intraoperative blood loss also varied between groups, with no clear correlation to SM grade, ranging from 263.5 (95% CI 164–363) in SM grade I + II to 273.5 ml (99–448) in SM grade III and 858.3 ml (95%CI 412.4–1304) in SM grade IV + V AVMs. Blood loss appeared to be greater in ruptured AVMs (901.8 ml, 95% CI 0–1937) compared with unruptured AVMs (332.6 ml, 95% CI 171.6–493.7). However, operative time appeared to be similar in patients presenting with ruptured bAVMs (332.9 min, 95% CI 213–452.5) compared with unruptured bAVMs (288.8 min, 95% CI 261–317).

## Discussion

Management of bAVMs is nuanced and complex. Optimal management remains a topical issue in current practice. Surgical excision, embolisation and stereotactic radiosurgery constitute the three pillars of contemporary treatment, being applied in isolation or in combination [2, 29]. Embolisation as a neo-adjuvant treatment to definitive surgical intervention has gained traction over the last 20 to 30 years. It is now commonly performed to assist microsurgical excision. This practice is purported to have several theoretical advantages, such as limiting blood loss, reducing operative time and decreasing risk of postsurgical haemorrhage caused by altered haemodynamic in surrounding normal parenchyma [4, 12]. Pasqualin et al. concluded that preoperative embolisation limits blood loss and reduces operative time [22]. However, a larger series by Donzelli et al. reported no significant difference in blood loss and longer operating time with preoperative embolisation [8]. A possible explanation for this is that the patients selected for preoperative embolisation in the latter study had larger bAVMs and more likely to have both superficial and deep drainage than the “surgery-only” group. DeMerritt et al. stated that preoperative embolisation “improves post-surgical outcomes” following a retrospective review of 30 patients treated with surgery and embolisation compared to 41 patients treated with surgery alone [7]. Despite demonstrating the group managed with preoperative embolisation to have improved postoperative Glasgow Outcome Scores, study groups were not matched in terms of SM grade and no breakdown of mode of presentation was provided (ruptured or unruptured). There remains a paucity of information in published literature pertaining to the application and outcomes of preoperative embolisation. Despite the practice becoming “well accepted and firmly established” [32], the early promise of this intervention has yet to be reproduced in larger series.

This systematic review illustrates several significant limitations in published literature to date. We found no randomised controlled trials (RCTs) comparing surgery with preoperative embolisation versus surgery alone. Most studies are retrospective with small sample sizes. Many studies were excluded from this review due to lack of clinical outcomes. Functional outcomes using standardised measures such as the modified Rankin score were rarely reported. Additionally, there is significant variability in the way outcomes are reported in different studies. Most studies evaluating complications associated with preoperative embolisation failed to correct for underlying confounders. Four of the studies included in this systematic review failed to break down outcomes according to SM grade of AVMs [9, 15, 28, 31]. Five studies failed to consider the impact of mode of presentation (haemorrhage vs other) on outcome [9, 15, 17, 19, 28].

Another finding of this systematic review is that complications specifically associated with preoperative embolisation occur in a significant number of patients. Total complications related to preoperative embolisation alone occurred in 29.4% (95% CI 19.6–40.2) of cases. Despite considerable variation in rates of “technical complications”, rates of complications associated with permanent neurological deficits were relatively consistent across included studies. We estimated that 6% (95% CI 3.9–8.5) of patients suffered from permanent deficits explicitly related to preoperative embolisation [9, 19]. Two studies reported mRS scores following embolisation, perhaps the most clinically relevant measure of outcome. In the study performed by Morgan et al., 3⋅37% of patients had mRS scores of > 2, whilst Weber et al. found 8.51% of patients to have mRS scores of > 2 following preoperative embolisation [19, 31]. Weber et al. performed the only study included in this systematic review which provided a patient-by-patient breakdown of mRS scores at time of admission, following embolisation and after surgery. The mRS of 21.3% (10/47) of these patients declined following embolisation, with 10.6% (5/47) developing an mRS > 2. A mortality rate of 1⋅02% (0–2.25%) directly attributed to embolisation was calculated across included studies [7, 17, 19, 20, 27, 28, 31].

The main aim of preoperative embolisation is to improve patient outcomes following microsurgical bAVM resection. In this systematic review, 16.5% (95% CI 8.2–26.7) of patients undergoing surgery following preoperative embolisation suffered from a complication related to the microsurgical procedure [17, 19, 20, 27], with 11.1% (95% CI 6.7–16.3) suffering a permanent neurological deficit [17, 19, 20, 27] and mortality rate of 1.3% (95% CI 0–4.4). In the absence of randomised controlled trials or at least prospective studies with a control group, it is difficult to make any conclusions from these studies. Currently, the only option is to compare these results with those of published reports of bAVM microsurgery without embolisation. Results in this systematic review are like published results of microsurgery alone. A recent cohort study by Schramm et al. which examined 288 patients undergoing microsurgical excision for bAVMs (the majority of which were treated with microsurgery alone, i.e. 244 patients) reported a gross total rate of permanent neurological deficits in 12.2% of patients and a mortality rate of 1.7% [25]. Fifty percent of patients in this study presented with intracranial haemorrhage, not dissimilar to the 41.28% of patients included in this review. The breakdown of SM grades in this study is like the grades included in our systematic review (SM I + II 58%, SM III 31%, SM IV + V 11% compared to 48%, 34% and 18%, respectively). It is noteworthy that half of included bAVMs in our analysis are low grade, in which the necessity of embolisation is questionable given that these lesions are generally amenable to excision alone. Given the substantial number of complications observed, the decision for preoperative embolisation must be individualised. The estimated combined risk of preop embolisation and surgery should be less than the estimated risk of surgery alone.

Other perceived benefits of preoperative embolisation include eased handling during surgery, reduced operative time and reduced blood loss [9, 28]. Unfortunately, the ease of handling bAVMs during surgery is subjective and difficult to measure and therefore rarely reported. In terms of blood loss and operative time, we only found four studies reporting these outcomes [17, 20, 27, 31]. Again, because these studies did not have a control arm (microsurgery alone group), no firm conclusions can be made regarding whether preoperative embolisation reduced operative time and/or blood loss.

This study represents the first systematic review in literature evaluating complications associated with preoperative embolisation of bAVMs. The quality of published reports on this topic are generally poor, with the majority being retrospective studies with no control arm (surgery alone) and paucity of data on SM grading, mode of presentation and objective outcomes (functional outcomes, operative time, blood loss and need for postoperative transfusion). Complication rates, including neurological disability and mortality associated with preoperative embolisation, are significant. Therefore, there is limited evidence to support the routine use of preoperative embolisation in the treatment of bAVMs. High-quality prospective studies and ideally randomised controlled trials are required to assess whether the claimed benefits of preoperative embolisation are realised and worth the risks associated with this intervention.

## Limitations

There are some limitations to this analysis. Firstly, all but one of the studies included in the analysis were retrospective cohort studies. This introduces potential biases; for example, centres with significant experience and large volume of endovascular procedures may selectively identify patients who would be suitable for preoperative embolisation better than smaller centres. As a meta-analysis of single-arm studies, it is important to note that these findings describe the risks associated with preoperative embolisation and subsequent surgical intervention but do not provide evidence of its relative efficacy or safety compared to surgical intervention alone. We observed substantial imprecision in many results, which reduces our confidence in the findings. Secondly there are inconsistent reports on the interval between embolisation and surgery, and whether or not this might contribute to the variations in intra-operative outcomes, i.e., blood loss, observed in our analysis. Finally, although the MEDLINE database is a robust database, it is possible that some non-indexed publications indexed in other databases may be omitted from our analysis.

## Conclusion

This meta-analysis identifies substantial risks associated with preoperative embolisation of bAVMs. The efficacy of preoperative embolisation is unclear given that no studies directly comparing patients undergoing excision with versus without embolisation have been published to date. Given this unclear efficacy in the context of a substantial risk of complications, there is currently insufficient evidence to support routine preoperative embolisation. Further studies (ideally randomised trials) comparing microsurgical excision of bAVMs with and without preoperative embolisation are warranted.

## Supplementary Information

Below is the link to the electronic supplementary material.

Supplementary file1 (PDF 62 kb)

## Data Availability

Data collected for the study will be made available upon request via email to the corresponding author. All data is available on request.
