# Supplementary material for: Preoperative embolisation of brain arteriovenous malformations: a systematic review and meta-analysis
Source: Neurosurg Rev. 2022 Mar 9;45(3):2051–63. doi: 10.1007/s10143-022-01766-8 (PMC9160113; doi:10.1007/s10143-022-01766-8)

Supplementary Figure 1

Preoperative embolisation of brain arteriovenous malformations. A systematic review of the literature.

Neurosurgical Review

Conor Brosnan MB, BCh*, Michael Amoo, MCh, Mohsen Javadpour, FRCS(SN)

***Corresponding author:**

Conor Brosnan
National Neurosurgical Centre, Beaumont Hospital, Dublin, 9
E: conorbrosnan@rcsi.ie

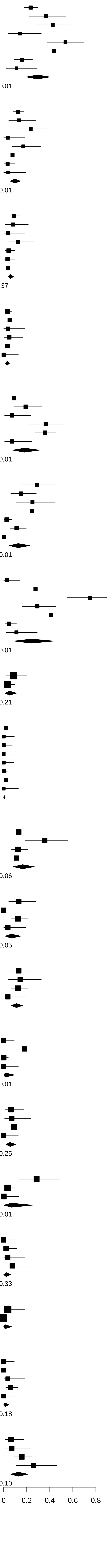

Study	Events	Total	Proportion	95%−CI
**embo_all**				
Taylor 2004	47	201	0.23	[0.18; 0.30]
Steiger 2004	14	38	0.37	[0.22; 0.54]
Weber 2007	20	47	0.43	[0.28; 0.58]
Natarajan 2008	4	28	0.14	[0.04; 0.33]
Hauck 2009	22	41	0.54	[0.37; 0.69]
Loh 2010	51	117	0.44	[0.34; 0.53]
Morgan 2013	14	89	0.16	[0.09; 0.25]
Luzzi 2018	3	27	0.11	[0.02; 0.29]
**Random effects model**	**175**	**588**	**0.29**	**[0.20; 0.40]**
Heterogeneity: $I^2 = 85\%$, $\tau^2 = 0.0207$, $p < 0.01$				
**embo_deficit**				
Taylor 2004	25	201	0.12	[0.08; 0.18]
Steiger 2004	5	38	0.13	[0.04; 0.28]
Weber 2007	11	47	0.23	[0.12; 0.38]
Natarajan 2008	1	28	0.04	[0.00; 0.18]
Hauck 2009	7	41	0.17	[0.07; 0.32]
Loh 2010	9	117	0.08	[0.04; 0.14]
Morgan 2013	3	89	0.03	[0.01; 0.10]
Luzzi 2018	1	27	0.04	[0.00; 0.19]
**Random effects model**	**62**	**588**	**0.10**	**[0.06; 0.15]**
Heterogeneity: $I^2 = 63\%$, $\tau^2 = 0.0062$, $p < 0.01$				
**embo_p_deficit**				
Taylor 2004	18	201	0.09	[0.05; 0.14]
Steiger 2004	3	38	0.08	[0.02; 0.21]
Natarajan 2008	1	28	0.04	[0.00; 0.18]
Hauck 2009	5	41	0.12	[0.04; 0.26]
Loh 2010	5	117	0.04	[0.01; 0.10]
Morgan 2013	3	89	0.03	[0.01; 0.10]
Luzzi 2018	1	27	0.04	[0.00; 0.19]
**Random effects model**	**36**	**541**	**0.06**	**[0.04; 0.08]**
Heterogeneity: $I^2 = 7\%$, $\tau^2 = 0.0003$, $p = 0.37$				
**embo_t_deficit**				
Taylor 2004	7	201	0.03	[0.01; 0.07]
Steiger 2004	2	38	0.05	[0.01; 0.18]
Natarajan 2008	1	28	0.04	[0.00; 0.18]
Hauck 2009	2	41	0.05	[0.01; 0.17]
Loh 2010	4	117	0.03	[0.01; 0.09]
Luzzi 2018	0	27	0.00	[0.00; 0.13]
**Random effects model**	**16**	**452**	**0.03**	**[0.01; 0.05]**
Heterogeneity: $I^2 = 0\%$, $\tau^2 = 0$, $p = 0.83$				
**embo_technical**				
Taylor 2004	18	201	0.09	[0.05; 0.14]
Weber 2007	9	47	0.19	[0.09; 0.33]
Natarajan 2008	2	28	0.07	[0.01; 0.24]
Hauck 2009	15	41	0.37	[0.22; 0.53]
Loh 2010	42	117	0.36	[0.27; 0.45]
Luzzi 2018	2	27	0.07	[0.01; 0.24]
**Random effects model**	**88**	**461**	**0.18**	**[0.08; 0.31]**
Heterogeneity: $I^2 = 89\%$, $\tau^2 = 0.0304$, $p < 0.01$				
**embo_haem**				
Steiger 2004	11	38	0.29	[0.15; 0.46]
Weber 2007	7	47	0.15	[0.06; 0.28]
Natarajan 2008	7	28	0.25	[0.11; 0.45]
Hauck 2009	10	41	0.24	[0.12; 0.40]
Loh 2010	3	117	0.03	[0.01; 0.07]
Morgan 2013	10	89	0.11	[0.06; 0.20]
Luzzi 2018	0	27	0.00	[0.00; 0.13]
**Random effects model**	**48**	**387**	**0.13**	**[0.05; 0.23]**
Heterogeneity: $I^2 = 85\%$, $\tau^2 = 0.0260$, $p < 0.01$				
**embo_nonhaem**				
Steiger 2004	1	38	0.03	[0.00; 0.14]
Weber 2007	13	47	0.28	[0.16; 0.43]
Natarajan 2008	21	28	0.75	[0.55; 0.89]
Hauck 2009	12	41	0.29	[0.16; 0.46]
Loh 2010	48	117	0.41	[0.32; 0.50]
Morgan 2013	4	89	0.04	[0.01; 0.11]
Luzzi 2018	3	27	0.11	[0.02; 0.29]
**Random effects model**	**102**	**387**	**0.24**	**[0.09; 0.44]**
Heterogeneity: $I^2 = 94\%$, $\tau^2 = 0.0711$, $p < 0.01$				
**embo_mrs**				
Weber 2007	4	47	0.09	[0.02; 0.20]
Morgan 2013	3	89	0.03	[0.01; 0.10]
**Random effects model**	**7**	**136**	**0.05**	**[0.01; 0.11]**
Heterogeneity: $I^2 = 37\%$, $\tau^2 = 0.0023$, $p = 0.21$				
**embo_mortality**				
Taylor 2004	4	201	0.02	[0.01; 0.05]
Steiger 2004	0	38	0.00	[0.00; 0.09]
Weber 2007	0	47	0.00	[0.00; 0.08]
Natarajan 2008	0	28	0.00	[0.00; 0.12]
Hauck 2009	0	41	0.00	[0.00; 0.09]
Loh 2010	0	117	0.00	[0.00; 0.03]
Morgan 2013	2	89	0.02	[0.00; 0.08]
Luzzi 2018	0	27	0.00	[0.00; 0.13]
**Random effects model**	**6**	**588**	**0.00**	**[0.00; 0.01]**
Heterogeneity: $I^2 = 0\%$, $\tau^2 = 0$, $p = 0.66$				
**surg_all**				
Steiger 2004	5	38	0.13	[0.04; 0.28]
Natarajan 2008	10	28	0.36	[0.19; 0.56]
Morgan 2013	11	89	0.12	[0.06; 0.21]
Luzzi 2018	3	27	0.11	[0.02; 0.29]
**Random effects model**	**29**	**182**	**0.16**	**[0.08; 0.27]**
Heterogeneity: $I^2 = 59\%$, $\tau^2 = 0.0089$, $p = 0.06$				
**surg_deficit**				
Steiger 2004	5	38	0.13	[0.04; 0.28]
Natarajan 2008	0	28	0.00	[0.00; 0.12]
Morgan 2013	11	89	0.12	[0.06; 0.21]
Luzzi 2018	1	27	0.04	[0.00; 0.19]
**Random effects model**	**17**	**182**	**0.07**	**[0.01; 0.15]**
Heterogeneity: $I^2 = 62\%$, $\tau^2 = 0.0101$, $p = 0.05$				
**surg_p_deficit**				
Steiger 2004	5	38	0.13	[0.04; 0.28]
Natarajan 2008	4	28	0.14	[0.04; 0.33]
Morgan 2013	11	89	0.12	[0.06; 0.21]
Luzzi 2018	1	27	0.04	[0.00; 0.19]
**Random effects model**	**21**	**182**	**0.11**	**[0.07; 0.16]**
Heterogeneity: $I^2 = 0\%$, $\tau^2 = 0$, $p = 0.54$				
**surg_t_deficit**				
Steiger 2004	0	38	0.00	[0.00; 0.09]
Natarajan 2008	5	28	0.18	[0.06; 0.37]
Morgan 2013	0	89	0.00	[0.00; 0.04]
Luzzi 2018	0	27	0.00	[0.00; 0.13]
**Random effects model**	**5**	**182**	**0.01**	**[0.00; 0.09]**
Heterogeneity: $I^2 = 79\%$, $\tau^2 = 0.0229$, $p < 0.01$				
**surg_haem**				
Weber 2007	3	47	0.06	[0.01; 0.18]
Natarajan 2008	2	28	0.07	[0.01; 0.24]
Morgan 2013	8	89	0.09	[0.04; 0.17]
Luzzi 2018	0	27	0.00	[0.00; 0.13]
**Random effects model**	**13**	**191**	**0.06**	**[0.02; 0.11]**
Heterogeneity: $I^2 = 27\%$, $\tau^2 = 0.0021$, $p = 0.25$				
**surg_nonhaem**				
Natarajan 2008	8	28	0.29	[0.13; 0.49]
Morgan 2013	3	89	0.03	[0.01; 0.10]
Luzzi 2018	0	27	0.00	[0.00; 0.13]
**Random effects model**	**11**	**144**	**0.07**	**[0.00; 0.25]**
Heterogeneity: $I^2 = 87\%$, $\tau^2 = 0.0421$, $p < 0.01$				
**surg_residual**				
Steiger 2004	0	38	0.00	[0.00; 0.09]
Weber 2007	1	47	0.02	[0.00; 0.11]
Natarajan 2008	1	28	0.04	[0.00; 0.18]
Luzzi 2018	2	27	0.07	[0.01; 0.24]
**Random effects model**	**4**	**140**	**0.02**	**[0.00; 0.06]**
Heterogeneity: $I^2 = 12\%$, $\tau^2 = 0.0010$, $p = 0.33$				
**surg_infection**				
Natarajan 2008	1	28	0.04	[0.00; 0.18]
Luzzi 2018	0	27	0.00	[0.00; 0.13]
**Random effects model**	**1**	**55**	**0.01**	**[0.00; 0.07]**
Heterogeneity: $I^2 = 0\%$, $\tau^2 = 0$, $p = 0.33$				
**surg_mortality**				
Steiger 2004	0	38	0.00	[0.00; 0.09]
Weber 2007	0	47	0.00	[0.00; 0.08]
Natarajan 2008	1	28	0.04	[0.00; 0.18]
Morgan 2013	5	89	0.06	[0.02; 0.13]
Luzzi 2018	0	27	0.00	[0.00; 0.13]
**Random effects model**	**6**	**229**	**0.01**	**[0.00; 0.04]**
Heterogeneity: $I^2 = 36\%$, $\tau^2 = 0.0032$, $p = 0.18$				
**surg_mrs**				
Weber 2007	3	47	0.06	[0.01; 0.18]
Natarajan 2008	2	28	0.07	[0.01; 0.24]
Morgan 2013	14	89	0.16	[0.09; 0.25]
Luzzi 2018	7	27	0.26	[0.11; 0.46]
**Random effects model**	**26**	**191**	**0.13**	**[0.06; 0.21]**
Heterogeneity: $I^2 = 53\%$, $\tau^2 = 0.0063$, $p = 0.10$				

Supplementary Figure 2

Preoperative embolisation of brain arteriovenous malformations. A systematic review of the literature

Neurosurgical Review

Conor Brosnan MB, BCh*, Michael Amoo, MCh, Mohsen Javadpour, FRCS(SN)

***Corresponding author:**
Conor Brosnan
National Neurosurgical Centre, Beaumont Hospital, Dublin, 9
E: conorbrosnan@rcsi.ie

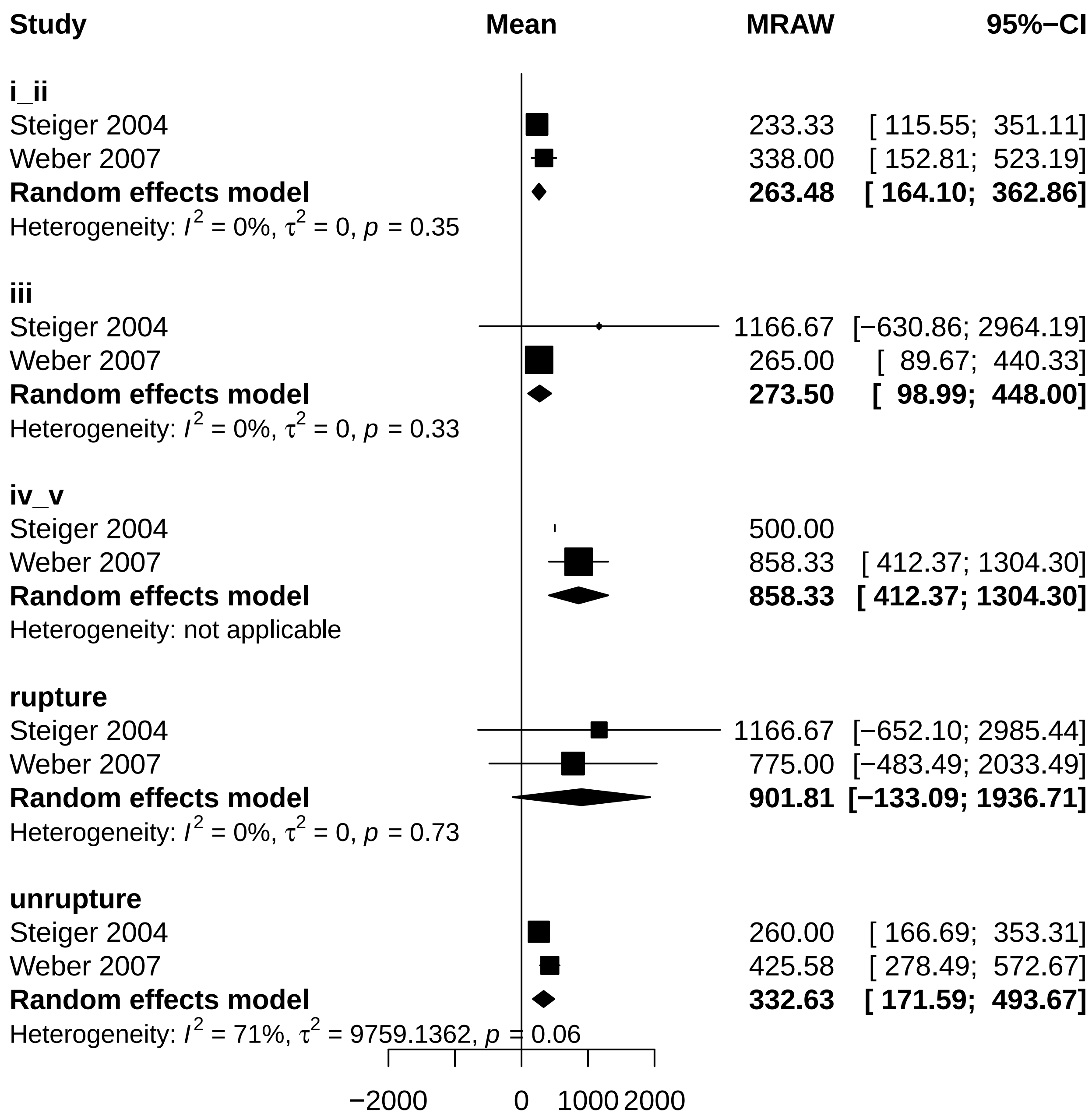

Supplementary Figure 3

Preoperative embolisation of brain arteriovenous malformations. A systematic review of the literature
Neurosurgical Review

Conor Brosnan MB, BCh*, Michael Amoo, MCh, Mohsen Javadpour, FRCS(SN)

***Corresponding author:**
Conor Brosnan
National Neurosurgical Centre, Beaumont Hospital, Dublin, 9
E: conorbrosnan@rcsi.ie

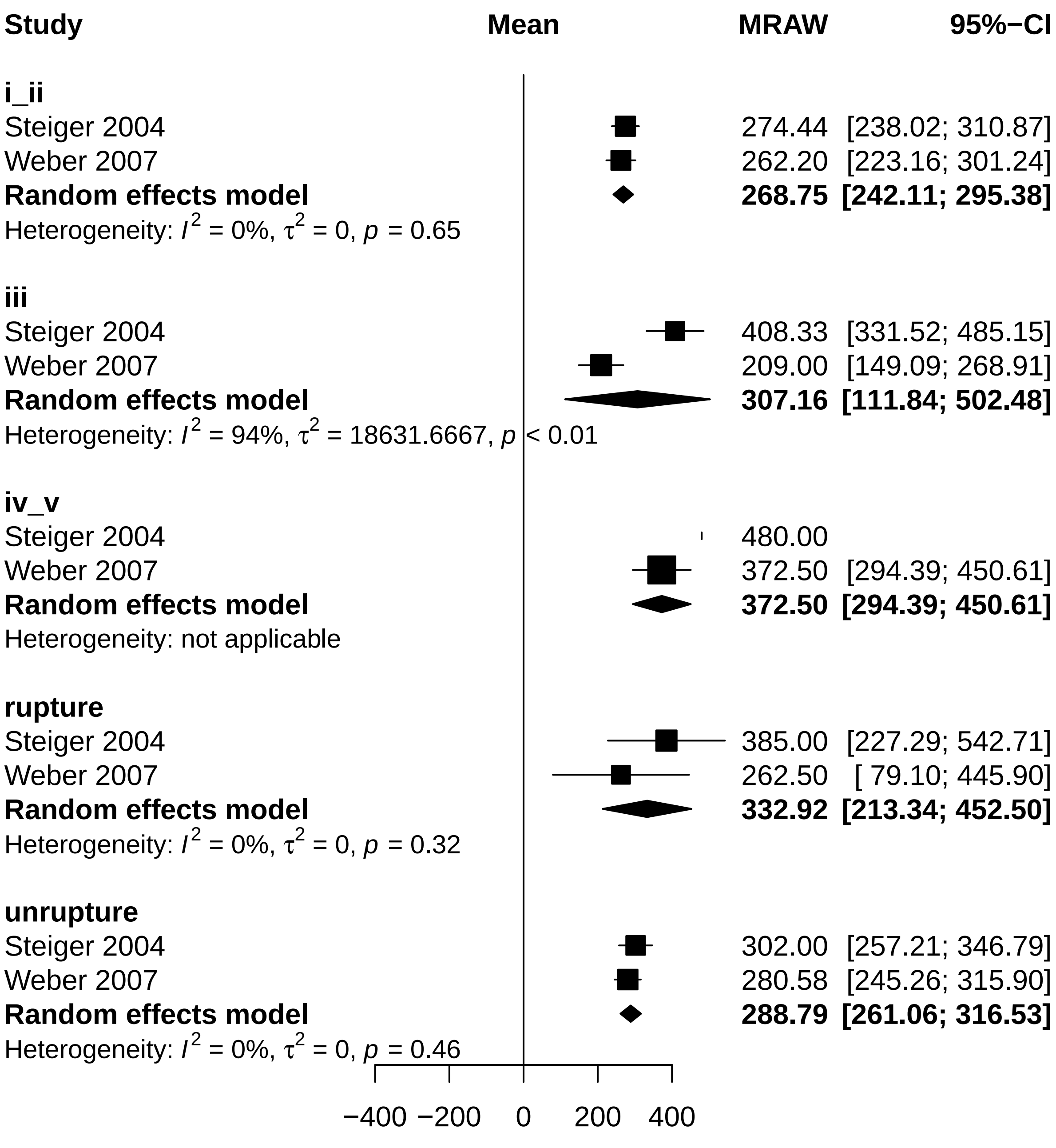

Supplementary Figure 4

Preoperative embolisation of brain arteriovenous malformations. A systematic review of the literature
Neurosurgical Review

Conor Brosnan MB, BCh*, Michael Amoo, MCh, Mohsen Javadpour, FRCS(SN)

***Corresponding author:**
Conor Brosnan
National Neurosurgical Centre, Beaumont Hospital, Dublin, 9
E: conorbrosnan@rcsi.ie

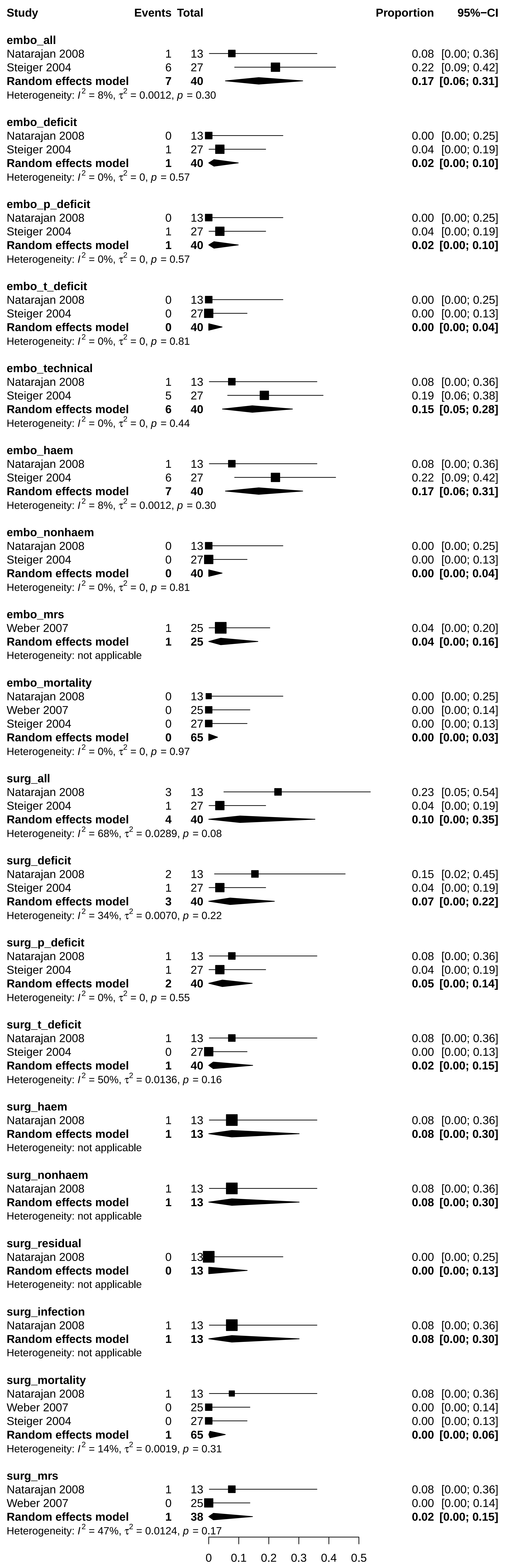

Supplementary Figure 5

Preoperative embolisation of brain arteriovenous malformations. A systematic review of the literature
Neurosurgical Review

Conor Brosnan MB, BCh*, Michael Amoo, MCh, Mohsen Javadpour, FRCS(SN)

***Corresponding author:**
Conor Brosnan
National Neurosurgical Centre, Beaumont Hospital, Dublin, 9
E: conorbrosnan@rcsi.ie

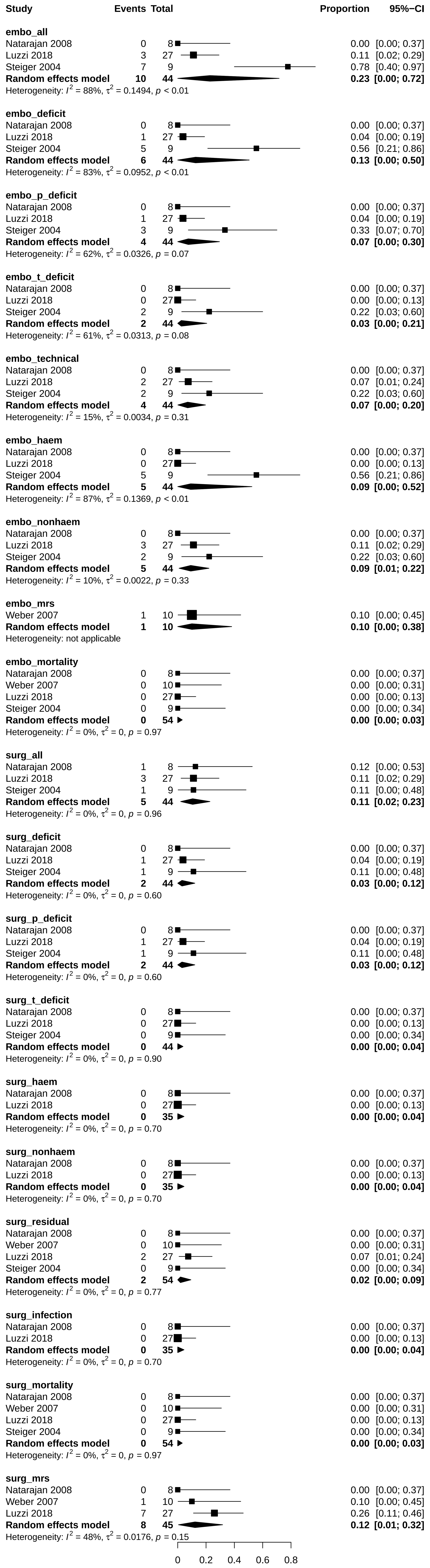

Supplementary Figure 6

Preoperative embolisation of brain arteriovenous malformations. A systematic review of the literature
Neurosurgical Review

Conor Brosnan MB, BCh*, Michael Amoo, MCh, Mohsen Javadpour, FRCS(SN)

*Corresponding author:
Conor Brosnan
National Neurosurgical Centre, Beaumont Hospital, Dublin, 9
E: conorbrosnan@rcsi.ie

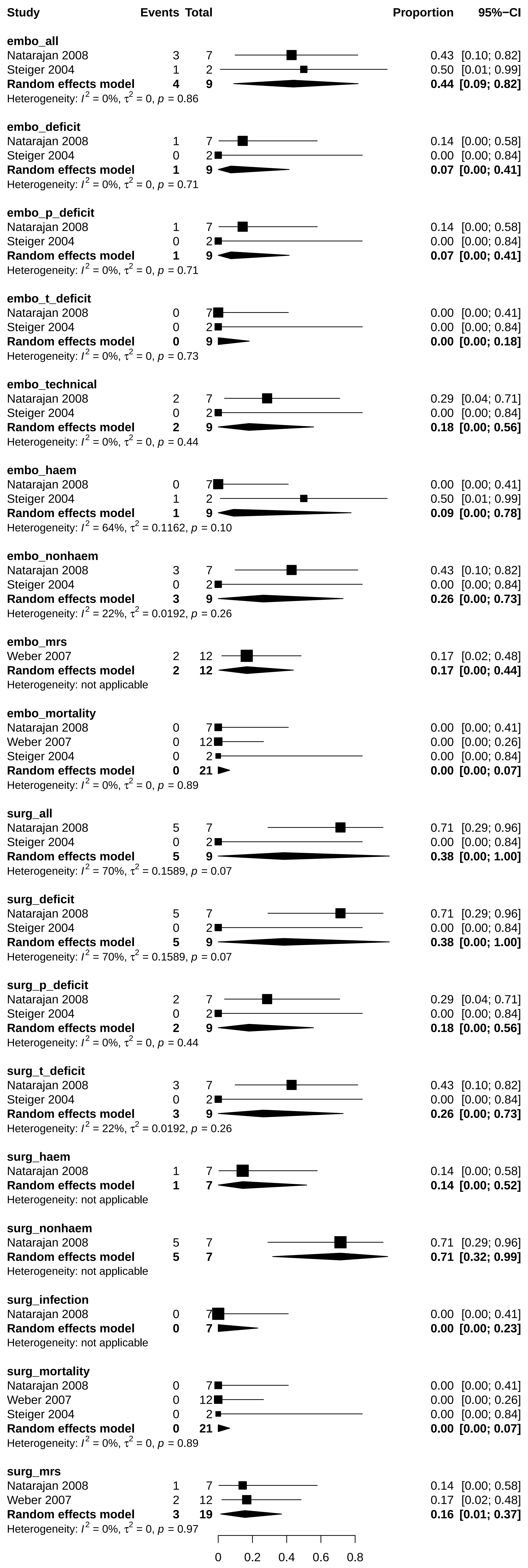

Supplementary Figure 7

Preoperative embolisation of brain arteriovenous malformations. A systematic review of the literature
Neurosurgical Review

Conor Brosnan MB, BCh*, Michael Amoo, MCh, Mohsen Javadpour, FRCS(SN)

*Corresponding author:
Conor Brosnan
National Neurosurgical Centre, Beaumont Hospital, Dublin, 9
E: conorbrosnan@rcsi.ie

Study	Events	Total	Proportion	95%-CI
**embo_all**				
Steiger 2004	4	14	0.29	[0.08; 0.58]
Natarajan 2008	2	14	0.14	[0.02; 0.43]
**Random effects model**	**6**	**28**	**0.21**	**[0.07; 0.39]**
Heterogeneity: $I^2 = 0\%$, $\tau^2 = 0$, $p = 0.39$				
**embo_deficit**				
Steiger 2004	2	14	0.14	[0.02; 0.43]
Natarajan 2008	0	14	0.00	[0.00; 0.23]
**Random effects model**	**2**	**28**	**0.05**	**[0.00; 0.26]**
Heterogeneity: $I^2 = 58\%$, $\tau^2 = 0.0243$, $p = 0.12$				
**embo_p_deficit**				
Steiger 2004	0	14	0.00	[0.00; 0.23]
Natarajan 2008	0	14	0.00	[0.00; 0.23]
**Random effects model**	**0**	**28**	**0.00**	**[0.00; 0.07]**
Heterogeneity: $I^2 = 0\%$, $\tau^2 = 0$, $p = 1.00$				
**embo_t_deficit**				
Steiger 2004	2	14	0.14	[0.02; 0.43]
Natarajan 2008	0	14	0.00	[0.00; 0.23]
**Random effects model**	**2**	**28**	**0.05**	**[0.00; 0.26]**
Heterogeneity: $I^2 = 58\%$, $\tau^2 = 0.0243$, $p = 0.12$				
**embo_technical**				
Steiger 2004	2	14	0.14	[0.02; 0.43]
Natarajan 2008	2	14	0.14	[0.02; 0.43]
**Random effects model**	**4**	**28**	**0.14**	**[0.03; 0.31]**
Heterogeneity: $I^2 = 0\%$, $\tau^2 = 0$, $p = 1.00$				
**embo_haem**				
Steiger 2004	2	14	0.14	[0.02; 0.43]
Natarajan 2008	0	14	0.00	[0.00; 0.23]
**Random effects model**	**2**	**28**	**0.05**	**[0.00; 0.26]**
Heterogeneity: $I^2 = 58\%$, $\tau^2 = 0.0243$, $p = 0.12$				
**embo_nonhaem**				
Steiger 2004	2	14	0.14	[0.02; 0.43]
Natarajan 2008	2	14	0.14	[0.02; 0.43]
**Random effects model**	**4**	**28**	**0.14**	**[0.03; 0.31]**
Heterogeneity: $I^2 = 0\%$, $\tau^2 = 0$, $p = 1.00$				
**embo_mrs**				
Weber 2007	1	4	0.25	[0.01; 0.81]
**Random effects model**	**1**	**4**	**0.25**	**[0.00; 0.79]**
Heterogeneity: not applicable				
**embo_mortality**				
Steiger 2004	0	14	0.00	[0.00; 0.23]
Weber 2007	0	4	0.00	[0.00; 0.60]
Natarajan 2008	0	14	0.00	[0.00; 0.23]
**Random effects model**	**0**	**32**	**0.00**	**[0.00; 0.05]**
Heterogeneity: $I^2 = 0\%$, $\tau^2 = 0$, $p = 0.92$				
**surg_all**				
Steiger 2004	1	14	0.07	[0.00; 0.34]
Natarajan 2008	4	14	0.29	[0.08; 0.58]
**Random effects model**	**5**	**28**	**0.17**	**[0.01; 0.41]**
Heterogeneity: $I^2 = 50\%$, $\tau^2 = 0.0170$, $p = 0.16$				
**surg_deficit**				
Steiger 2004	1	14	0.07	[0.00; 0.34]
Natarajan 2008	3	14	0.21	[0.05; 0.51]
**Random effects model**	**4**	**28**	**0.14**	**[0.02; 0.30]**
Heterogeneity: $I^2 = 0\%$, $\tau^2 = 0$, $p = 0.32$				
**surg_p_deficit**				
Steiger 2004	1	14	0.07	[0.00; 0.34]
Natarajan 2008	1	14	0.07	[0.00; 0.34]
**Random effects model**	**2**	**28**	**0.07**	**[0.00; 0.21]**
Heterogeneity: $I^2 = 0\%$, $\tau^2 = 0$, $p = 1.00$				
**surg_t_deficit**				
Steiger 2004	0	14	0.00	[0.00; 0.23]
Natarajan 2008	2	14	0.14	[0.02; 0.43]
**Random effects model**	**2**	**28**	**0.05**	**[0.00; 0.26]**
Heterogeneity: $I^2 = 58\%$, $\tau^2 = 0.0243$, $p = 0.12$				
**surg_haem**				
Steiger 2004	0	14	0.00	[0.00; 0.23]
Natarajan 2008	1	14	0.07	[0.00; 0.34]
**Random effects model**	**1**	**28**	**0.02**	**[0.00; 0.13]**
Heterogeneity: $I^2 = 1\%$, $\tau^2 = 0.0002$, $p = 0.31$				
**surg_nonhaem**				
Steiger 2004	0	14	0.00	[0.00; 0.23]
Natarajan 2008	3	14	0.21	[0.05; 0.51]
**Random effects model**	**3**	**28**	**0.07**	**[0.00; 0.39]**
Heterogeneity: $I^2 = 75\%$, $\tau^2 = 0.0522$, $p = 0.04$				
**surg_residual**				
Steiger 2004	0	14	0.00	[0.00; 0.23]
Natarajan 2008	0	14	0.00	[0.00; 0.23]
**Random effects model**	**0**	**28**	**0.00**	**[0.00; 0.07]**
Heterogeneity: $I^2 = 0\%$, $\tau^2 = 0$, $p = 1.00$				
**surg_infection**				
Steiger 2004	0	14	0.00	[0.00; 0.23]
Natarajan 2008	0	14	0.00	[0.00; 0.23]
**Random effects model**	**0**	**28**	**0.00**	**[0.00; 0.07]**
Heterogeneity: $I^2 = 0\%$, $\tau^2 = 0$, $p = 1.00$				
**surg_mortality**				
Steiger 2004	0	14	0.00	[0.00; 0.23]
Weber 2007	0	4	0.00	[0.00; 0.60]
Natarajan 2008	1	14	0.07	[0.00; 0.34]
**Random effects model**	**1**	**32**	**0.01**	**[0.00; 0.11]**
Heterogeneity: $I^2 = 0\%$, $\tau^2 = 0$, $p = 0.60$				
**surg_mrs**				
Weber 2007	1	4	0.25	[0.01; 0.81]
Natarajan 2008	1	14	0.07	[0.00; 0.34]
**Random effects model**	**2**	**18**	**0.09**	**[0.00; 0.30]**
Heterogeneity: $I^2 = 0\%$, $\tau^2 = 0$, $p = 0.34$				

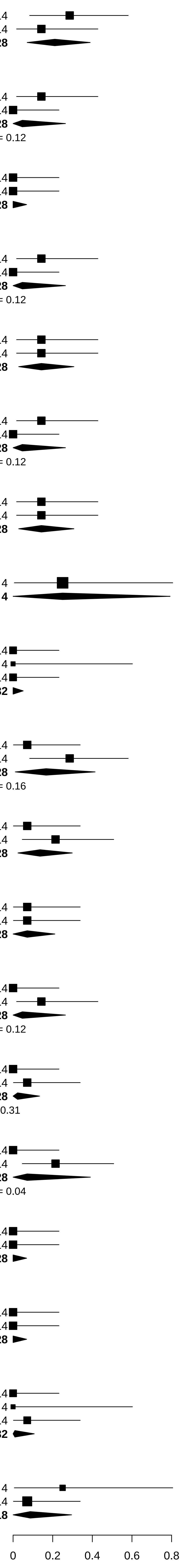

Supplementary Figure 8

Preoperative embolisation of brain arteriovenous malformations. A systematic review of the literature
Neurosurgical Review

Conor Brosnan MB, BCh*, Michael Amoo, MCh, Mohsen Javadpour, FRCS(SN)

***Corresponding author:**
Conor Brosnan
National Neurosurgical Centre, Beaumont Hospital, Dublin, 9
E: conorbrosnan@rcsi.ie

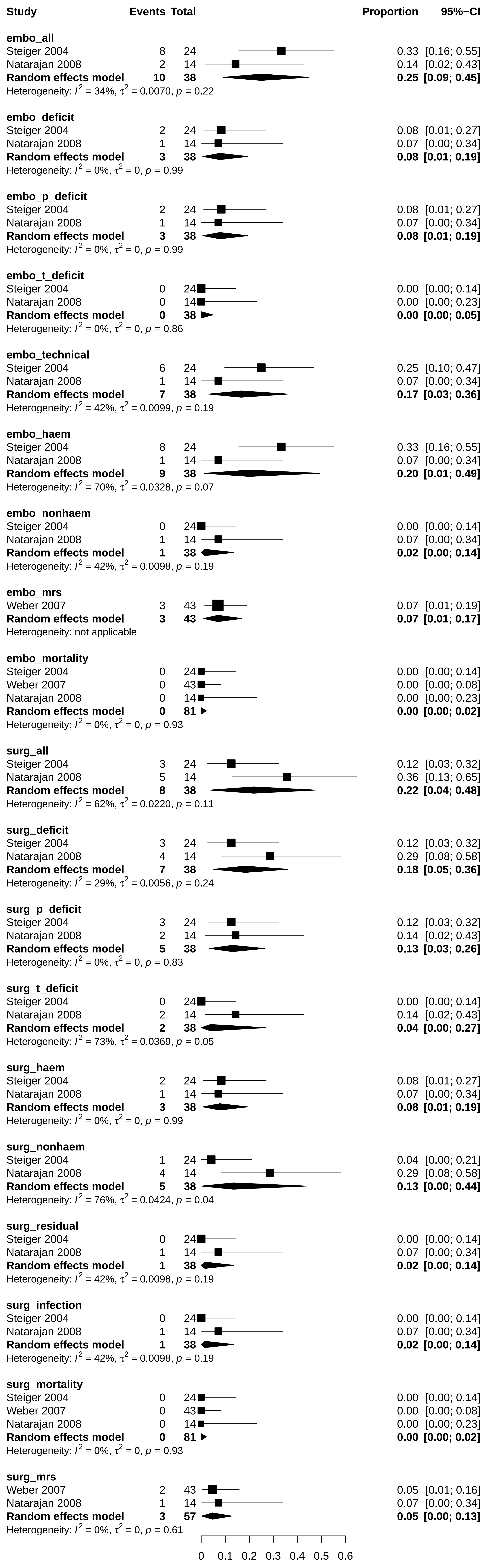

Supplement: Supplementary file 1 — Supplementary file1 (PDF 62 kb) [file 10143_2022_1766_MOESM1_ESM.pdf]
